# SARS-CoV-2 enzyme-linked immunosorbent assays as proxies for plaque reduction neutralisation tests

**DOI:** 10.1038/s41598-022-07263-8

**Published:** 2022-03-01

**Authors:** Grant A. Kay, Sophie I. Owen, Emanuele Giorgi, David J. Clark, Christopher T. Williams, Stefanie Menzies, Luis E. Cuevas, Benedict M. O. Davies, Nicholas M. Eckersley, Grant L. Hughes, Daniela E. Kirwan, Sanjeev Krishna, Edward I. Patterson, Tim Planche, Henry M. Staines, Emily R. Adams

**Affiliations:** 1 Centre for Drugs and Diagnostics, Liverpool School of Tropical Medicine, Liverpool, UK; 2 Departments of Vector Biology and Tropical Disease Biology, Centre for Neglected Tropical Diseases, Liverpool School of Tropical Medicine, Liverpool, UK; 3 Centre for Health Informatics Computing and Statistics, Lancaster University Medical School, Lancaster University, Lancaster, UK; 4 Centre for Diagnostics & Antimicrobial Resistance, Clinical Academic Group in Institute for Infection & Immunity, St George’s University of London, London, UK; 5 St George’s University Hospitals NHS Foundation Trust, London, UK; 6 Institut für Tropenmedizin, Universitätsklinikum Tübingen, Tübingen, Germany; 7 Centre de Recherches Médicales de Lambaréné, Lambaréné, Gabon; 8 Department of Biological Sciences, Brock University, St. Catharines, Canada

**Keywords:** Diagnostic markers, Viral infection, SARS-CoV-2, Antibodies

## Abstract

Severe acute respiratory coronavirus 2 (SARS-CoV-2) has spread globally since its emergence in 2019. Most SARS-CoV-2 infections generate immune responses leading to rising levels of immunoglobulins (Ig) M, A and G which can be detected using diagnostic tests including enzyme-linked immunosorbent assays (ELISA). Whilst implying previous SARS-CoV-2 infection, the detection of Ig by ELISA does not guarantee the presence of neutralising antibodies (NAb) that can prevent the virus infecting cells. Plaque reduction neutralisation tests (PRNT) detect NAb, but are not amenable to mass testing as they take several days and require use of SARS-CoV-2 in high biocontainment laboratories. We evaluated the ability of IgG and IgM ELISAs targeting SARS-CoV-2 spike subunit 1 receptor binding domain (S1-RBD), and spike subunit 2 (S2) and nucleocapsid protein (NP), at predicting the presence and magnitude of NAb determined by PRNT. IgG S2 + NP ELISA was 96.8% [95% CI 83.8–99.9] sensitive and 88.9% [95% CI 51.8–99.7] specific at predicting the presence of NAbs (PRNT_80_ > 1:40). IgG and IgM S1-RBD ELISAs correlated with PRNT titre, with higher ELISA results increasing the likelihood of a robust neutralising response. The IgM S1-RBD assay can be used as a rapid, high throughput test to approximate the magnitude of NAb titre.

## Introduction

Severe acute respiratory syndrome coronavirus 2 (SARS-CoV-2) is a novel, pandemic betacoronavirus that began spreading globally in early 2020. To date, there have been over 198 million reported infections and more than 4.2 million deaths^1^.

Most individuals infected with SARS-CoV-2 develop humoral immune responses, characterised by rising titres of immunoglobulins (Ig) M, A and G, within the first 2–3 weeks of infection^2,3^, which are detectable using enzyme-linked immunosorbent assays (ELISA). The presence of SARS-CoV-2 specific Ig therefore provides evidence of previous infection^4^, although their detection does not guarantee the presence of functional immunity against the virus^5^. For example, the viral nucleocapsid protein (NP), an abundant viral antigen, generates robust antibody responses, and is therefore a good antigen for diagnostic serological assays^6^, however these antibodies are not neutralising^7,8^.

SARS-CoV-2 neutralising antibodies (NAb) primarily bind the receptor-binding domain of the spike (S) protein and disrupt virus entry by blocking interaction with the angiotensin converting enzyme 2 (ACE2) receptor of host cells^7,9^. The activity of these functional antibodies can be measured using the plaque reduction neutralisation test (PRNT). However, this method is not amenable to mass testing, as the process takes several days and requires working with SARS-CoV-2 in high biocontainment laboratories. Pseudotyped virus models may be used to detect neutralising antibodies under reduced biocontainment, but still lack the speed and reproducibility of ELISA assays^10^.

Previous studies have reported that NAb levels correlate with IgG and IgM titres^11–14^, but this relationship is variable, depending on the timing of sampling in the course of the infection and the antigen targets of the serological assays^15^. Here we evaluate the ability of SARS-CoV-2 IgG and IgM ELISAs to predict the presence and magnitude of SARS-CoV-2 NAbs in hospitalised COVID-19 patients.

## Methods

### Ethical statement

The study was conducted in accordance with relevant UK guidelines and regulations. Ethics approval was provided by the Institutional Review Board (South Central—Oxford C Research Ethics Committee, Research Development and Assessment of Rapid Testing for SARS-CoV-2 outbreak study; Integrated Research Application System project ID:282104; Research Ethics Committee Reference 20/SC/0171; registered at clinicaltrial.gov NCT04351646). The approved protocol permitted the analysis of antibody responses using anonymised excess diagnostic material (EDM) from the pathology laboratory of patients with and without PCR-confirmed SARS-CoV-2 infection. Informed consent was not required under the ethical approval status of the work and due to the nature of the samples.

### Serum samples

Anonymized EDM serum samples from hospital patients with SARS-CoV-2 infection confirmed by reverse transcription—quantitative polymerase chain reaction (RT-qPCR) were used for this study and were selected from 645 EDM serum samples that were collected from a pool of 177 patients treated at St George’s Hospital, London UK^16^. Where possible, samples were selected from patients at least 10 days post-RT-qPCR confirmation. Samples were grouped based on their normalised optical density (NOD) values derived from an anti-SARS-CoV-2 IgG S2 and NP ELISA carried out previously^16^. These were grouped into “negative NOD” values (< 0; indicating the patient had not seroconverted), “low NOD” (0 to 0.5), “medium NOD” (0.9 to 1.1); and “high NOD” (> 1.5). The final sample available from all patients was chosen for this study. The narrow “medium NOD” window was purposely selected to reduce sample numbers in this grouping, as the grouping 0.5 to 1.5 contained 5–6 times more samples than the other groupings. A single sample was then selected from any patient with at least 3 samples with NOD values remaining in one NOD grouping (i.e. indicating a stable antibody response). The serum sample selected for any given patient was that collected furthest from the swab taken for confirmation of SARS-CoV-2 infection (and at least 10 days post-swab). The approach resulted in 9, 9, 11 and 12 patient samples in each group (41 single patient serum samples in total).

All participants were confirmed as positive for SARS-CoV-2 using RT-PCR from nose/throat swabs (in Sigma Virocult®, Corsham, UK) and Roche RNA extraction kits (Magnapure, West Sussex, UK) followed by Altona Diagnostics RealStar® SARS-CoV-2 RT-PCR (S and E target genes, Hamburg, Germany) or Roche cobas® SARS-CoV-2 Test (E and ORF target genes).

### ELISA to detect anti-SARS-CoV-2 IgM binding S2 and NP antigens

An anti-SARS-CoV-2 IgM ELISA (Mologic, Bedfordshire, UK), which targets the NP and S2 antigens, were used to measure antibodies, as per the manufacturer’s instructions. Briefly, sera were diluted (1:200) and incubated on a S2/NP pre-coated plate (30 min) at room temperature and then washed three times. Conjugated antibody (anti-human IgM) was then applied to each well and incubated (30 min) at room temperature. Following washing (× 4), TMB substrate was added and incubated for 10 min at room temperature before addition of stop solution. Optical densities (OD) were read at 450 nm within 10 min of addition of the stop solution.

### ELISA to detect anti-SARS-CoV-2 IgG binding the receptor binding domain of S1 (S1-RBD)

An anti-SARS-CoV-2 IgG ELISA (Abcam, Cambridgeshire, UK), which targets S1-RBD antigen, was used to measure human antibodies, as per the manufacturer’s instructions. Briefly, samples (sera diluted 1 in 100), a calibrator (Recombinant Human anti-RBD IgG), positive control (Recombinant Human anti-RBD IgG) and negative control (diluent) were added to an S1-RBD pre-coated plate. The plate was sealed with film and incubated (30 min) at room temperature and then washed five times. HRP Conjugated antibody (Goat Anti-Human IgG-Fc) was then applied to each, the plate sealed and incubated (30 min) at room temperature. Following washing (× 5), TMB substrate was added and the plate incubated for 15 min in the dark at room temperature before addition of stop solution. Optical densities (OD) were read at 450 nm and 570 nm within 10 min of addition of the stop solution.

### ELISA to detect anti-SARS-CoV-2 IgM binding the receptor binding domain of S1 (S1-RBD)

An anti-SARS-CoV-2 IgM ELISA (Abcam, Cambridgeshire, UK), which targets S1-RBD antigen, was also used to measure human antibodies, following the same procedure but with HRP conjugated Goat Anti-Human IgM-Fc antibody and Recombinant Human anti-RBD IgM controls.

### Plaque reduction neutralisation tests

Vero E6 cells were seeded into 24-well cell culture plates at a density of 250,000 cells/ml and incubated (24 h, 37 °C, 5% CO_2_). The following day serum samples were heated to inactivate complement (56 °C for 1 h). Heat-inactivated serum samples were twofold serially diluted in infection media (DMEM with 2% v/v FBS and 1:1000 50 mg/ml gentamicin). Under biosafety level 3 conditions, SARS-CoV-2 isolate REMRQ0001/Human/2020/Liverpool^17^ was added to an equal volume of diluted patient serum, at a titre of 800 pfu/ml, to achieve 12 final serum dilutions from 1:20 to 1:40,960 for each patient sample. Following incubation (1 h, 37 °C), the virus-serum mixture (100 µl) was inoculated onto Vero E6 cells and incubated (1 h, 37 °C, 5% CO_2_) before applying an overlay of infection media containing agarose (0.4% w/v). Infected cells were then incubated (48 h, 37 °C, 5% CO_2_). The assays were fixed with formaldehyde (37% w/v), stained with crystal violet solution (0.25% w/v) and allowed to air dry. The PRNT_80_ was determined as the lowest dilution of serum that produced a ≥ 80% reduction in the number of plaques compared to controls that contained no patient serum. The investigators were blinded to the ELISA status of the samples when performing the PRNTs.

### Western blots

Western blots were conducted to investigate the antigen binding profiles associated with the neutralising responses. Recombinant S1, S2 and NP (Native Antigen Company, Kidlington, UK) were prepared in NuPAGE LDS sample buffer (Invitrogen, Carlsbad, USA) with 50 mM dithiothreitol (Sigma, St Louis, USA), and heated for 10 min at 70 °C. Protein (1 µg) was loaded on a Mini-PROTEAN TGX 12% PAGE gel (BioRad, Hercules, USA) and run under reducing conditions in Tris/Glycine/SDS Buffer (BioRad, Hercules, USA). Proteins were transferred to 0.2 µm nitrocellulose membrane using BioRad TransBlot Turbo system mixed MW programme, and blocked in 5% (v/v) goat serum (Sigma, St Louis, USA) in PBS (Gibco, Waltham, USA) with 0.1% (v/v) Tween 20 (Sigma, St Louis, USA) overnight at 4 °C with gentle shaking. Blots were incubated with sera diluted 1 in 200 in blocking solution for 1 h at room temperature with gentle shaking and washed three times for 5 mins with PBS-0.1% (v/v) Tween 20. Blots were then incubated with goat anti-human Kappa-AP and anti-human Lambda-AP (Southern Biotech, Birmingham, USA), both diluted 1 in 1000 in blocking solution, for 1 h at room temperature with gentle shaking, followed by three washes as above. Blots were developed with BCIP/NBT (Sigma, St Louis, USA) for 1 min and stopped with H_2_O. The assays were scored visually as positive or negative by two independent observers who were blinded to the ELISA status of the samples. In the event of a disagreement between observers, a third independent observer determined the result. One IgG ELISA negative sample was excluded because there was not enough serum remaining for western blot.

### Statistical analysis

We model the PRNT80 outcome for the i-th patient, denoted by ${Y}_{i}$, as an ordinal variable taking the values from k = 1, corresponding to a titre less than 1:20, to k = 10, for a titre of 1:2560 or more. We assume that the observed titre values correspond to the discretization of a continuous antibody distribution, ${Y}_{i}^{*}$, which we assume to follow a log-Gaussian distribution with mean ${\vartheta }_{i}$ and variance ${\sigma }^{2}$.

Hence, we can write:

1 $$P({Y}_{i}=k)=P({{a}_{k}{<Y}_{i}^{*}<{a}_{k+1}}), k=1,...,10,$$

where ${a}_{1}=0$, ${a}_{2}=20$, ${a}_{3}=40$ and so forth up ${a}_{10}=2560$ and with the convention ${a}_{11}=\infty$. The probability in (1) is computed using the cumulative density function of a log-Gaussian distribution.

We model the mean concentration ${\vartheta }_{i}$ of PRNT80 as a log-linear regression on: ${a}_{i}$, a covariate which defined as IgG S1-RBD, IgM S1-RBD, IgG S2 + NP and IgM S1 + NP, in four different fitted models; and the days post-symptom, ${b}_{i}$. Hence, the linear predictor for ${\vartheta }_{i}$ is:

$$log\left\{{\vartheta }_{i}\right\}={\alpha }+{\beta }{a}_{i}+{\gamma b}_{i}$$

where the ${\alpha }$ is the intercept; ${\beta }$ express the strength of the selected antibody concentrations on the log-mean levels of PRNT_80_; similarly, $\gamma$ indicates the effect of the sampling time days post-symptom onset. All four models are fitted via maximum likelihood using the R software environment (Supplementary Data 1). Since the variable days post-symptom was not found to be significant at the conventional 95% confidence level, Tables 1 and 2 show the estimates from the best fitting models according to the likelihood values, after excluding that variable.

**Table 1:** Maximum likelihood estimates for the models for IgG S1-RBD, after excluding days post-onset of symptom variable.

Parameter	Estimate	95% confidence interval
${\alpha }$	5.317	(4.35, 6.285)
${\beta }$	0.187	(0.112, 0.262)
${\sigma }$	3277.827	(846.694, 12,689.540)

**Table 2:** Maximum likelihood estimates for the models for IgM S1-RBD, after excluding days post-onset of symptom variable.

Parameter	Estimate	95% confidence interval
${\alpha }$	5.800	(5.029, 6.572)
${\beta }$	0.454	(0.302, 0.607)
${\sigma }$	2278.542	(619.139, 8385.442)

## Results

### Patient demographics and symptoms

Twenty-five (61.0%) of the 41 patients were male, and the median age was 63 (IQR 55–71) years. Seventeen patients (41.5%) were classified as white, 13 (31.7%) non-white, and 11 (26.8%) were of unknown or ‘other’ ethnicity. Twenty-three patients (56.1%) had one or more comorbidities. Ten patients (24.4%) were obese, with a body mass index (BMI) > 30. These patients come from a subset already described^16^.

Of the thirty-nine patients for whom data were available, 35 (89.7%) were symptomatic at the time of their initial swab. Thirty-three of these patients (94.3%) had one or more of the classic triad of symptoms: cough, fever, and shortness of breath, 12 (34.3%) had gastrointestinal symptoms, including nine patients with diarrhoea. Three patients (7.3%) had an incidental positive swab, taken prior to admission to a rehabilitation facility, and three patients were swabbed following contact with a patient who had tested positive. The median interval between the onset of symptoms and date of the first positive swab was four days (IQR 3–7 days). Seven (17%) patients died within 28 days of their first positive swab. The median timing of serum sampling post symptom onset was 29 days (range 13–60 days). The sample timing post-symptom onset was not available for six patients.

### Diagnostic sensitivity of ELISAs at identifying a PRNT_80_ ≥ 1:40

**Figure 1:**
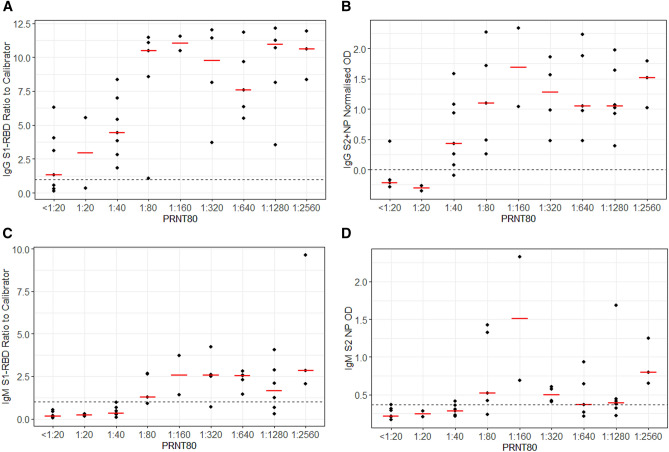
PRNT_80_ by IgG S1-RBD ratio to calibrator (**A**); IgM S1-RBD ratio to calibrator (**B**); IgG S2 + NP NOD_450_ (**C**); and IgM S2 + NP OD_450_ (**D**). Median values are indicated by the red horizontal bars. The cut-off used to determine a positive sample by each ELISA is indicated by the dashed horizontal line.

The correlation of IgG and IgM ELISAs and NAb responses are shown in Fig. 1. The IgG S1-RBD and IgG S2 + NP ELISAs identified a PRNT_80_ ≥ 1:40 with 100% [95% CI 89.1–100.0]) and 96.8% [95% CI 83.8–99.9] sensitivity, respectively (Table 3). This was higher than either of the IgM ELISAs (IgM S1-RBD sensitivity 62.5% [95% CI 43.7–78.9]; IgM S2 + NP sensitivity 62.5% [95% CI 43.7–78.9]). However, the IgG S1-RBD ELISA demonstrated poor specificity (44.4% [95% CI 13.7–78.8]) compared to the IgM S1-RBD (100.0% [95% CI 66.4–100.0]), IgG S2 + NP 88.9% [95% CI 51.8–99.7]), and IgM S2 + NP ELISAs (88.9% [95% CI 51.8–99.7]).

**Table 3:** Sensitivity and specificity of IgG and IgM ELISAs at identifying a PRNT_80_ titre of ≥ 1:40.

ELISA	Sensitivity (%) [95% CI]	Specificity (%) [95% CI]
IgG S1-RBD	100.0 [89.1–100.0]	44.4 [13.7–78.8]
IgM S1-RBD	62.5 [43.7–78.9]	100.0 [66.4–100.0]
IgG S2 + NP	96.8 [83.8–99.9]	88.9 [51.8–99.7]
IgM S2 + NP	62.5 [43.7–78.9]	88.9 [51.8–99.7]

Four samples that were only ELISA positive by the IgG S1-RBD assay, did not exhibit a neutralising response (PRNT ≥ 1:40). In addition, one sample was positive by both IgG S1-RBD, and IgG S2 + NP ELISA but did not show a neutralising response (PRNT_80_ ≥ 1:40). Both IgM ELISAs were negative for this sample. One non-neutralising sample (PRNT_80_ < 1:40) was only positive by IgM S2 + NP ELISA. One sample that had a PRNT_80_ of 1:40 was negative by all ELISAs except IgG S1-RBD. Several samples with neutralising responses (PRNT_80_ ≥ 1:40) were negative by both IgM ELISAs, but positive by both IgG assays.

### Correlation of ELISA and PRNT

Separate ordinal outcomes models were fitted to explore the relationship of each of the IgG and IgM ELISAs, and the timing of serum sampling post-onset of symptoms (denoted $\gamma$) on the observed neutralising titre (PRNT_80_). The maximum likelihood estimates from these models are shown in Tables 4, 5, 6 and 7. The IgG S1-RBD, IgM S1-RBD and IgG S2 + NP ELISAs were significant predictors of PRNT_80_ (0.191 [95% CI 0.117–0.266], 0.507 [95% CI 0.337–0.676], and 0.690 [95% CI 0.393–0.986], respectively). The IgM S2 + NP ELISA result was not a significant predictor of PRNT_80_ (− 0.053 [95% CI − 0.873–0.768]). The value of the likelihood functions for each of these models show that the IgG S1-RBD (-78.338) and IgM S1-RBD (− 77.414) assays best predicted PRNT_80_ (Table 8). The IgG and IgM S1-RBD maximum likelihood estimates, after excluding the non-significant $\gamma$ variable, indicated that each unit increase in antibody titre by ELISA was associated with a 0.187 [95% CI 0.112–0.262], and 0.454 [95% CI 0.302–0.607)-fold increased likelihood of being in a higher PRNT_80_ category, respectively.

**Table 4:** Maximum likelihood estimates and 95% confidence intervals for the model with IgG S1-RBD.

Parameter	Estimate	95% confidence interval
${\alpha }$	5.011	(3.864, 6.159)
${\beta }$	0.191	(0.117, 0.266)
$\gamma$	0.016	(− 0.017, 0.048)
${\sigma }$	3050.819	(816.476, 11,399.600)

**Table 5:** Maximum likelihood estimates and 95% confidence intervals for the model with IgM S1-RBD.

Parameter	Estimate	95% confidence interval
${\alpha }$	5.337	(4.353, 6.321)
${\beta }$	0.507	(0.337, 0.676)
$\gamma$	0.022	(− 0.009, 0.053)
${\sigma }$	2037.614	(592.384, 7008.743)

**Table 6:** Maximum likelihood estimates and 95% confidence intervals for the model with IgG S2 + NP.

Parameter	Estimate	95% confidence interval
${\alpha }$	6.255	(5.246, 7.263)
${\beta }$	0.690	(0.393, 0.986)
$\gamma$	− 0.002	(− 0.033, 0.030)
${\sigma }$	4724.139	(894.565, 24,947.880)

**Table 7:** Maximum likelihood estimates and 95% confidence intervals for the model with IgM S2 + NP.

Parameter	Estimate	95% confidence interval
${\alpha }$	7.231	(5.728, 8.734)
${\beta }$	− 0.053	(− 0.873, 0.768)
$\gamma$	0.020	(− 0.016, 0.056)
${\sigma }$	18,404.750	(1459.303, 232,120.800)

**Table 8:** Value of the likelihood function for each of the 4 models presented in Tables 4, 5, 6 and 7.

IgG S1-RBD	IgM S1-RBD	IgG S2 + NP	IgM S2 + NP
− 78.338	− 77.414	− 81.148	− 90.549

### Predicted PRNT_80_ based on IgM S1-RBD and IgG S1-RBD ELISA

**Figure 2:**
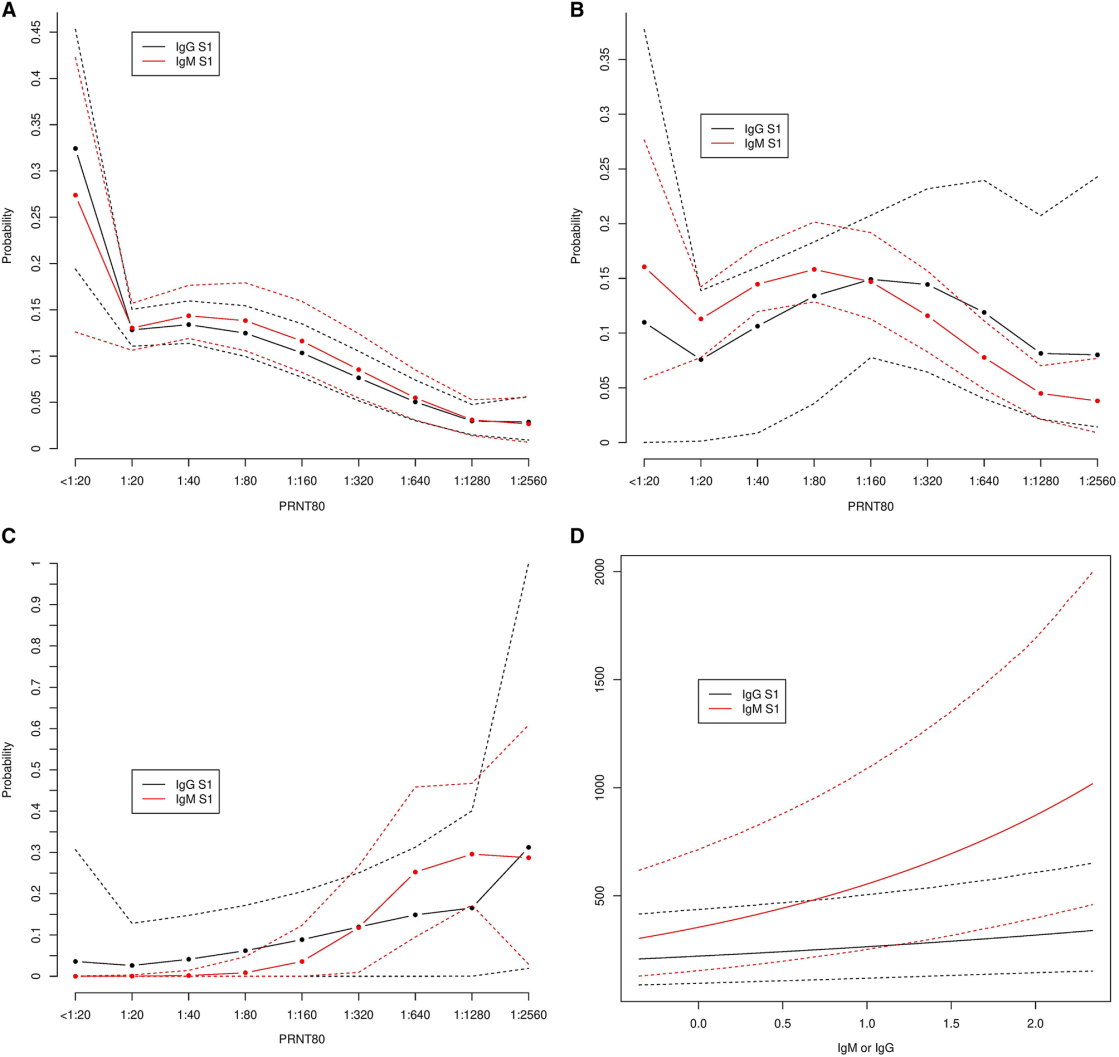
PRNT_80_ probability estimates of PRNT_80_ for first quartile (**A**); median (**B**); and third quartile IgG and IgM S1-RBD ELISA results (**C**). (**D**) shows predicted PRNT_80_ (NAb) by IgG or IgM S1-RBD ELISA result.

It was assumed that the relationship between IgM and IgG S1-RBD titres, and PRNT_80_ was unlikely to be linear across all the ranges of observed ELISA results. Therefore, the model predictions for PRNT_80_ based on IgG and IgM S1-RBD were considered for the first quartile, median, and third quartile of the antibody titres as measured by ELISA (see Fig. 2). For the first quartile, there was a low probability (< 0.15) of observing neutralising titres ≥ 1:20 for both IgM and IgG S1-RBD (Fig. 2A). For median titres, the highest probability (0.15) was for PRNT_80_ values in the range of 1:80–1:160 (Fig. 2B). Model predictions for IgG and IgM S1-RBD ELISA results in the third quartile indicated that PRNT_80_ values ≥ 1:320 were most likely to be observed for both IgG and IgM S1-RBD ELISAs (Fig. 2C).

Across the range of IgG S1-RBD titres detected by ELISA, we predicted that there was a low probability of observing an increasing PRNT_80_ value with increasing IgG S1-RBD, with predicted PRNT_80_ values remaining low despite increasing antibody binding detected by ELISA (Fig. 2D). IgM S1-RBD ELISA result demonstrated a stronger predicted quantifiable relationship with PRNT_80,_ albeit with increasing uncertainty at higher IgM S1-RBD ELISA results (Fig. 2D).

**Figure 3:**
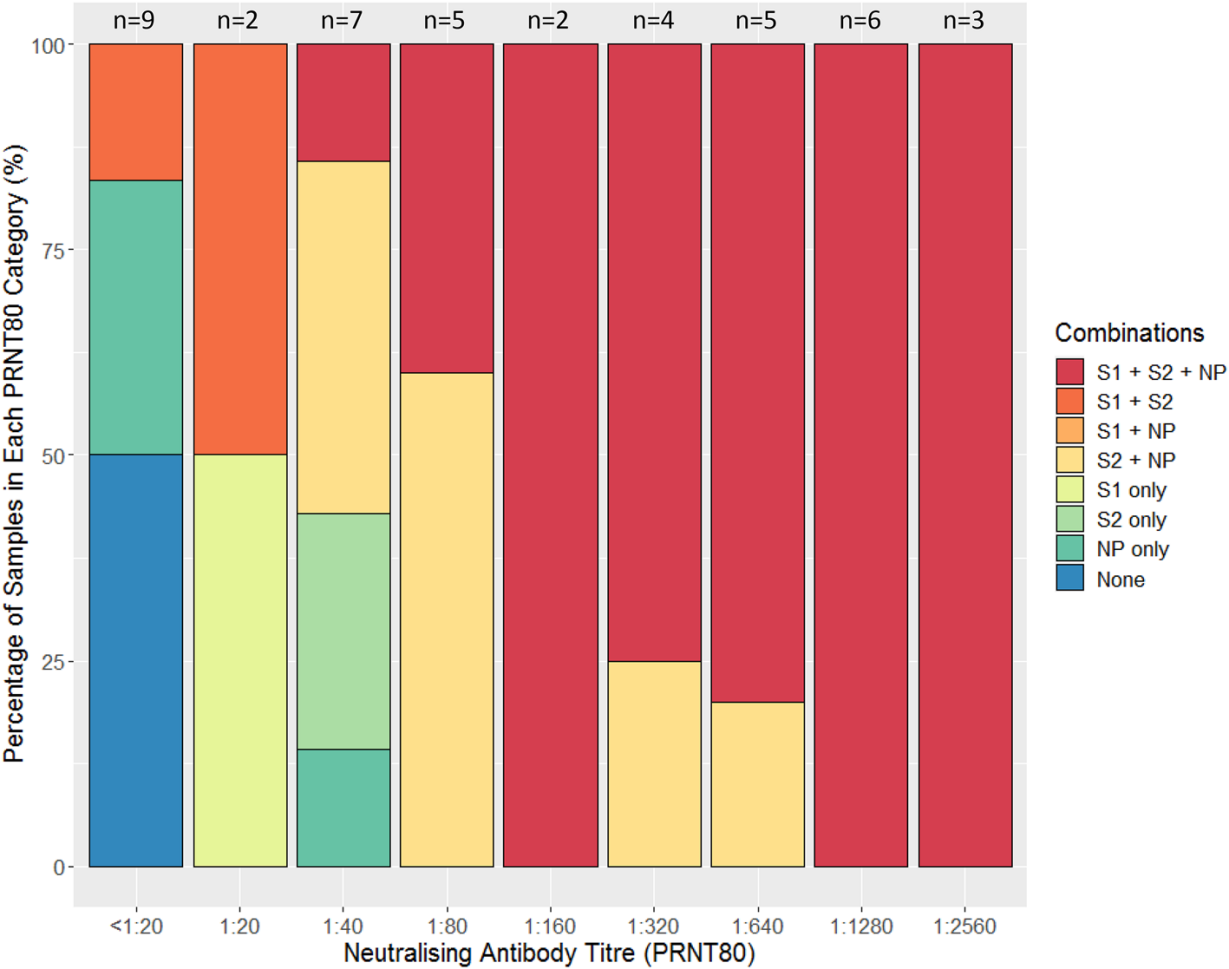
Percentage of samples in PRNT_80_ categories binding to different antigen combinations by Western Blot. *S1* spike protein subunit 1, *S2* spike protein subunit 2, *NP* nucleocapsid protein.

Antibody binding in western blot assays to all three of the S1, S2 and NP antigens, or to both the S2 and NP antigens, were seen in all samples that had high neutralising titres (PRNT_80_ ≥ 1:80) (Fig. 3). In samples with low PRNT_80_ (< 1:40) there was greater variability in antigen binding, with a larger proportion demonstrating antibody binding to single antigens, or combinations of two antigens involving S1. Three of the six samples that did not achieve PRNT_80_ ≥ 1:20 demonstrated a lack of binding to S1, S2 or NP. The full suite of ELISA, PRNT and Western blot results for each serum sample are available in Supplementary Data 2. Images of all Western blots are included in Supplementary Data 3.

## Discussion

Our data show that the IgG S2 + NP ELISA provides a sensitive (96.8%) and specific (88.9%) test to predict the presence of SARS-CoV-2 neutralising antibodies (PRNT_80_ ≥ 1:40). This is an interesting finding given that antibodies directed against S2 and NP are not considered to be highly neutralising^8^. However, NP-only IgG ELISAs have performed similarly to those targeting only the S1 antigen in previous evaluations^18,19^. This suggests that anti-NP antibodies are raised as part of a broad immune response, and whilst not directly neutralising, are indicative of the presence of other neutralising immunoglobulins. This is supported by our western blot analysis which revealed that the majority of serum samples with neutralising activity had antibodies directed against the NP, S1 and S2.

The IgG S1-RBD ELISA was also highly sensitive (100%) but was much less specific (44.4%). Our findings show there was a substantial chance of receiving a positive IgG S1-RBD ELISA result when there was no corresponding neutralising activity. Both IgG ELISAs were more sensitive than the IgM S1-RBD (62.5%) and IgM S2-RBD (62.5%) assays. However, the IgM S1-RBD ELISA demonstrated high specificity (100.0%). Previously, IgM ELISAs have been reported to be more predictive of neutralising titres than IgG, but given the short duration of IgM expression, the timing of serum sampling post-infection is an important determinant of these relationships^14,20,21^.

Our ordinal outcomes model demonstrated that both IgM S1-RBD and IgG S1-RBD ELISAs had the strongest correlation with PRNT_80_. Whilst our findings suggest that there is an increased probability of being in a higher PRNT_80_ category with increasing ELISA IgM or IgG S1-RBD titre, there was considerable variation in ELISA titre within each PRNT_80_ category. Consequently, it is not possible to make accurate predictions on the magnitude of NAb response based on the IgG S1-RBD ELISA. The model predictions indicate that IgM S1-RBD ELISA is the best predictor of the scale of the neutralising response, but there is significant uncertainty, especially at higher ELISA titres. Previous studies have reported a significant correlation between ELISAs targeting anti-spike and anti-RBD antigens of both IgG and IgM subclasses with the neutralisation titres established by microneutralisation tests, PRNTs and pseudotyped virus neutralisation assays^6,15,20,22–24^. However, all have noted a similar broad range of ELISA antibody titres within each neutralisation category.

The main limitation of our study is the small sample size. As the relationship between serological assays and neutralising titres appears to be variable, a larger sample size would have sufficient power to reveal overall trends and minimise the effects of outliers. IgA ELISA results have been reported to correlate well with neutralisation titres^24^. As we did not measure IgA, it is possible that neutralising activity due to IgA had a confounding effect on the trends observed. Moreover, our cohort only includes hospitalised patients with severe COVID-19 and therefore the findings of this study may not be applicable to individuals with mild and asymptomatic infections, or those who have been vaccinated against SARS-CoV-2, in whom antibody responses may be different.

In conclusion, ELISAs can be used as a proxy for PRNT to both predict the presence, and estimate the magnitude, of a neutralising antibody response. IgG ELISA targeting S2 + NP provides a sensitive and specific test to determine the presence of NAb. Whilst accurate predictions of precise PRNT_80_ values based on IgM S1-RBD ELISA are not possible, broad inferences on the magnitude of the neutralising response can be made using this assay. Assessment of NAb titre by PRNT is the gold standard and should be performed where feasible. However, given the biosafety requirements and low throughput nature of this technique, there is likely to be benefit in using more rapid and high throughput ELISAs to obtain approximations of NAb titre.

## Supplementary Information

Supplementary Information 1.

Supplementary Information 2.

Supplementary Information 3.
