# Supplementary material for: SARS-CoV-2 enzyme-linked immunosorbent assays as proxies for plaque reduction neutralisation tests
Source: Sci Rep. 2022 Mar 1;12:3351. doi: 10.1038/s41598-022-07263-8 (PMC8888744; doi:10.1038/s41598-022-07263-8)

SARS-CoV-2 enzyme-linked immunosorbent assays as proxies for plaque reduction neutralisation tests

Supplementary Data 3
Western blot images

Supplementary Data 3 – Western blot images
S1 = spike subunit 1, S2 = spike subunit 2, N = nucleocapsid

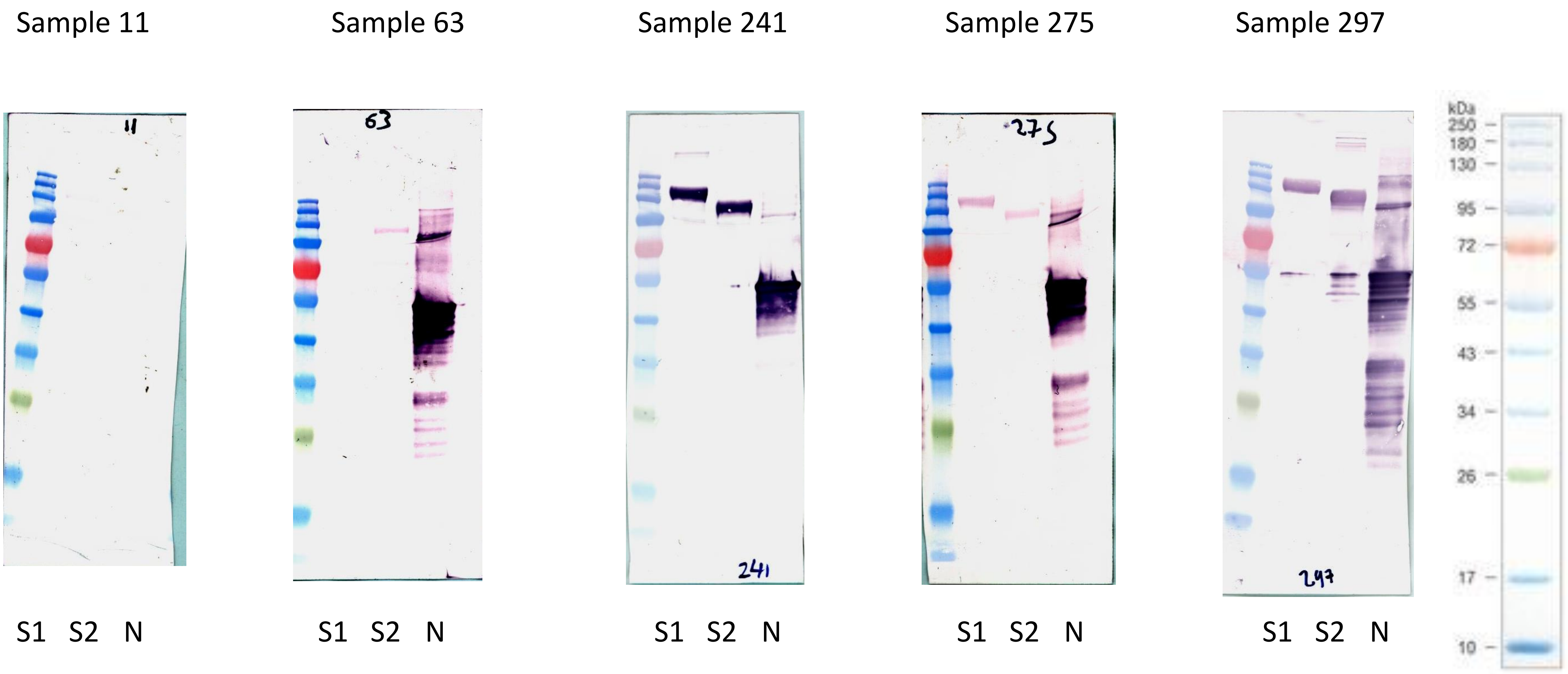

Supplementary Data 3 – Western blot images
S1 = spike subunit 1, S2 = spike subunit 2, N = nucleocapsid

Sample 303

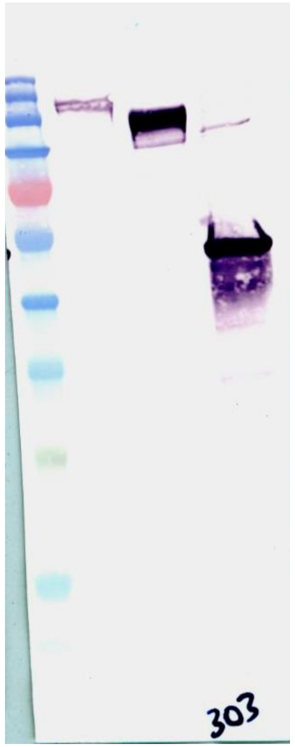

S1 S2 N

Sample 314

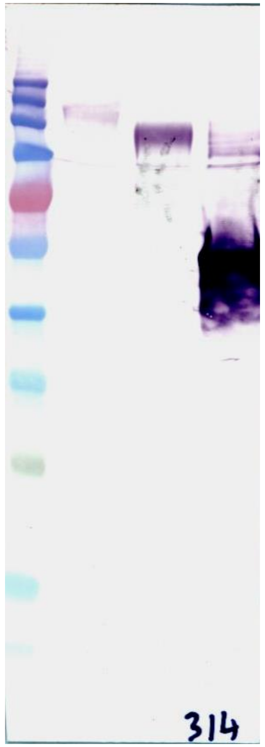

S1 S2 N

Sample 351

Insufficient sample volume for assay

Sample 355

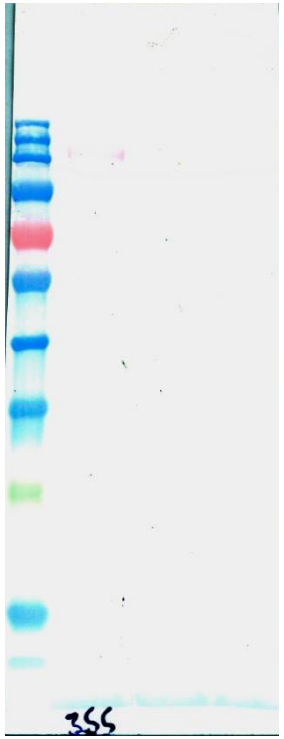

S1 S2 N

Sample 383

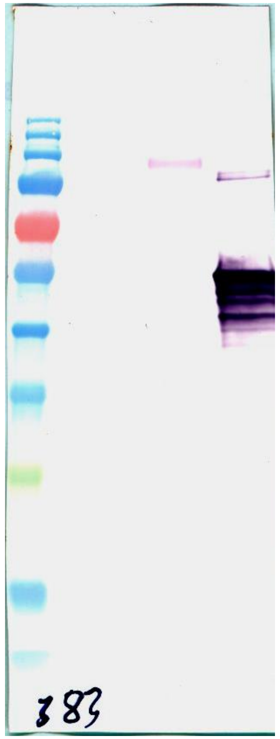

S1 S2 N

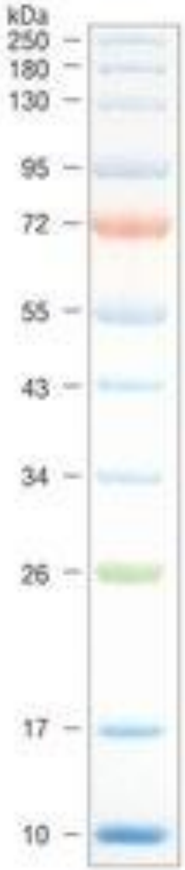

Supplementary Data 3 – Western blot images
S1 = spike subunit 1, S2 = spike subunit 2, N = nucleocapsid

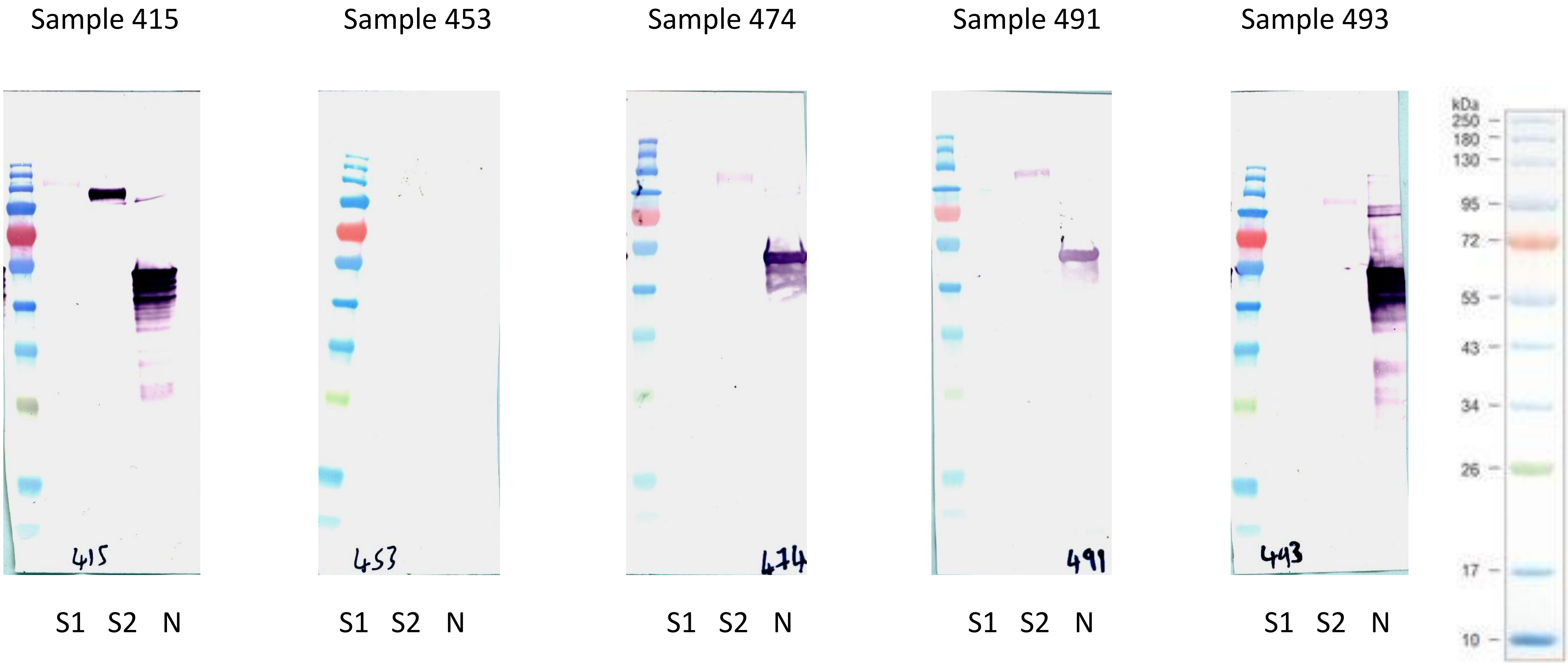

Supplementary Data 3 – Western blot images

S1 = spike subunit 1, S2 = spike subunit 2, N = nucleocapsid

Sample 497 Sample 515 Sample 545 Sample 551 Sample 566

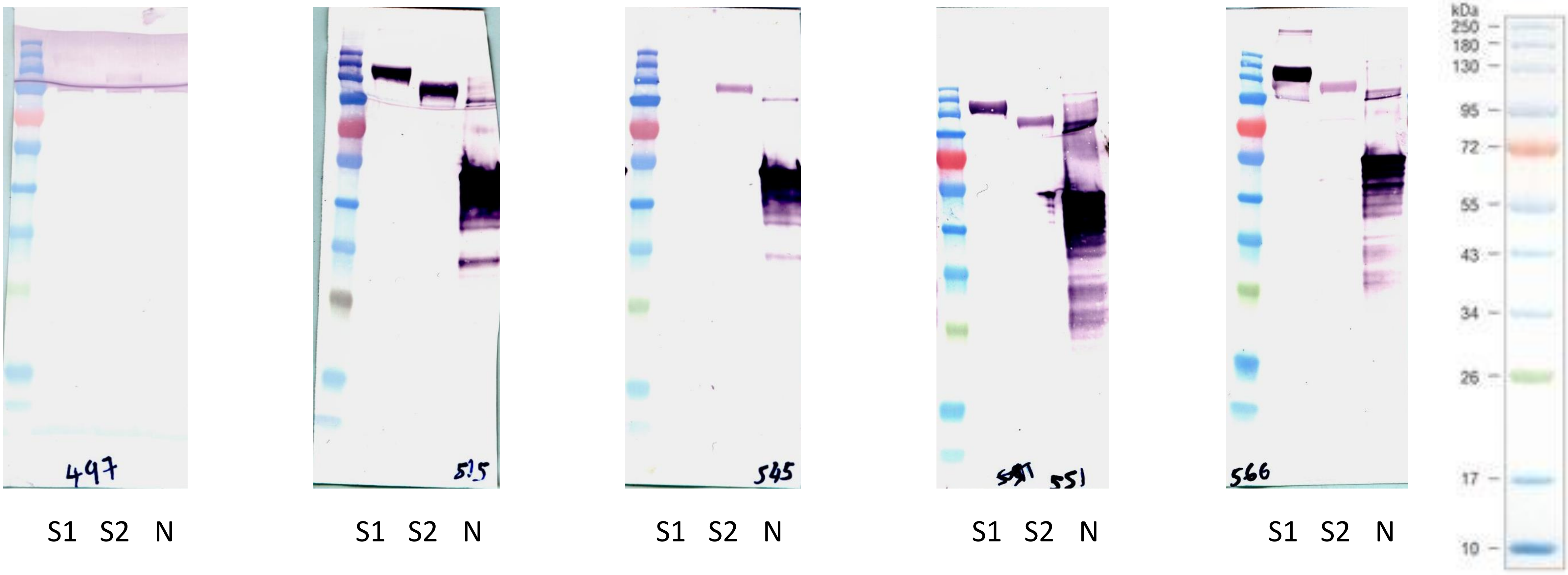

Supplementary Data 3 – Western blot images
S1 = spike subunit 1, S2 = spike subunit 2, N = nucleocapsid

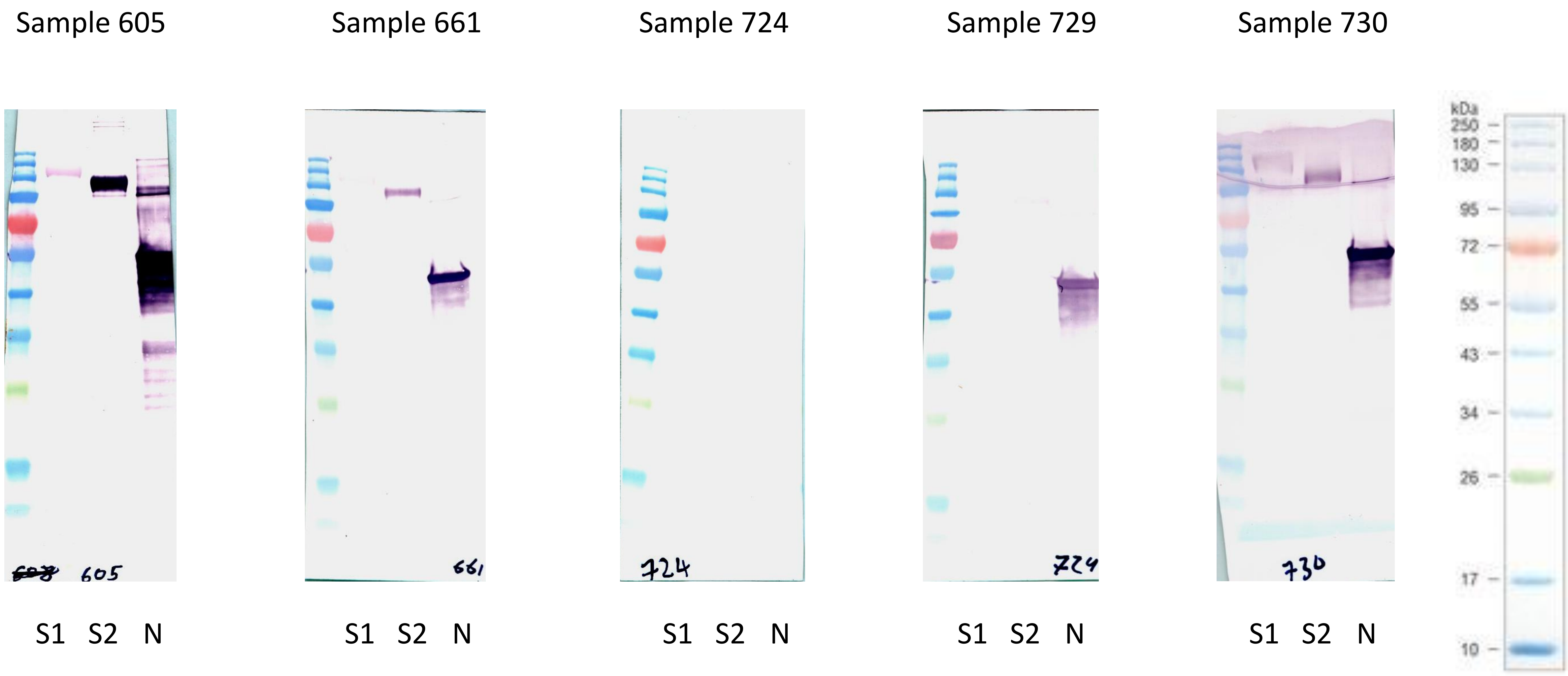

Supplementary Data 3 – Western blot images

S1 = spike subunit 1, S2 = spike subunit 2, N = nucleocapsid

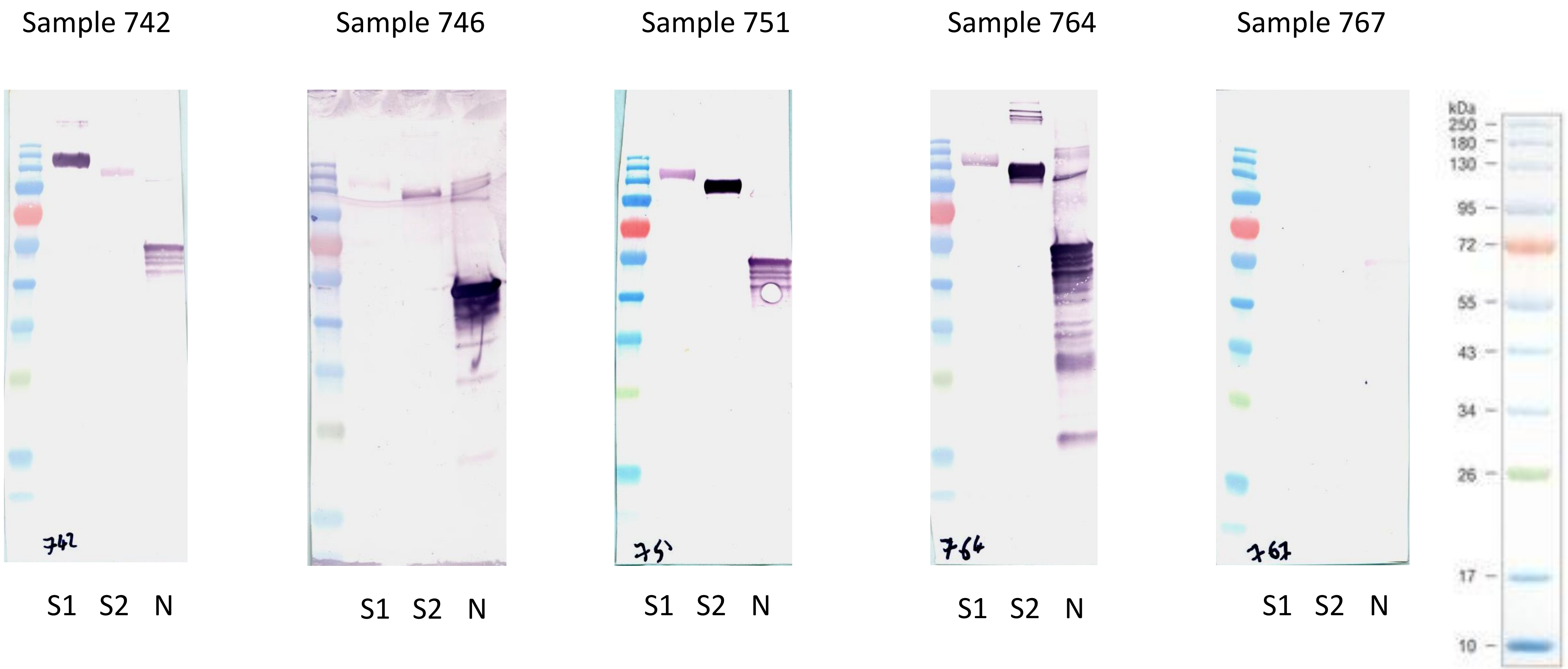

Supplementary Data 3 – Western blot images

S1 = spike subunit 1, S2 = spike subunit 2, N = nucleocapsid

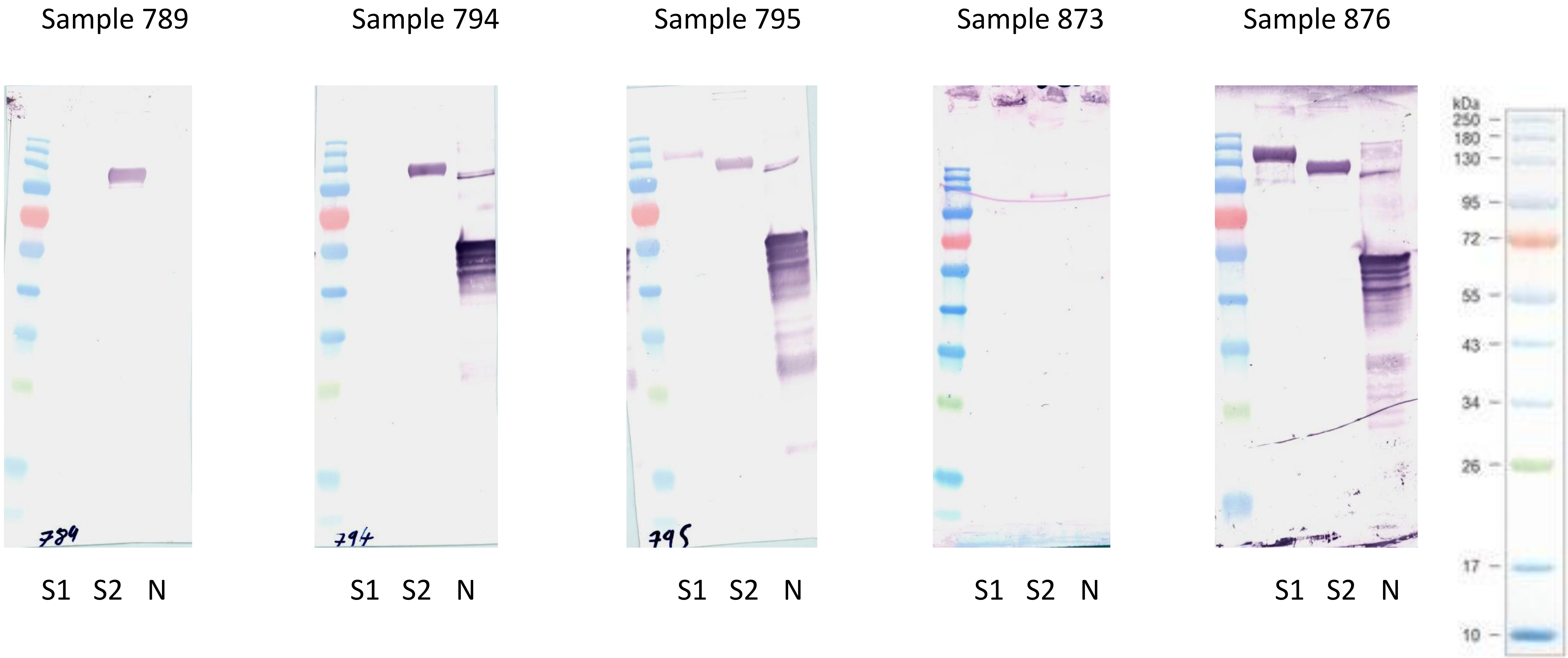

Supplementary Data 3 – Western blot images

S1 = spike subunit 1, S2 = spike subunit 2, N = nucleocapsid

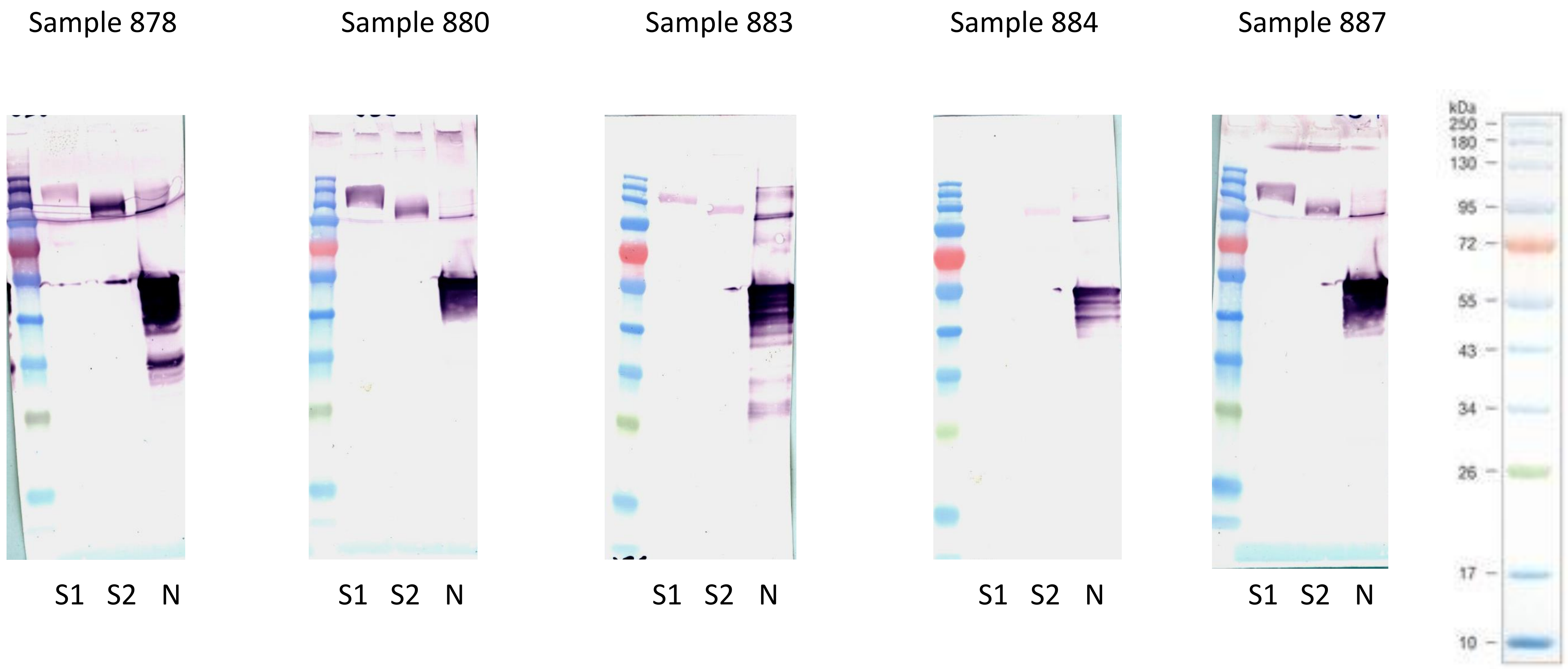

Supplementary Data 3 – Western blot images

S1 = spike subunit 1, S2 = spike subunit 2, N = nucleocapsid

Sample 888

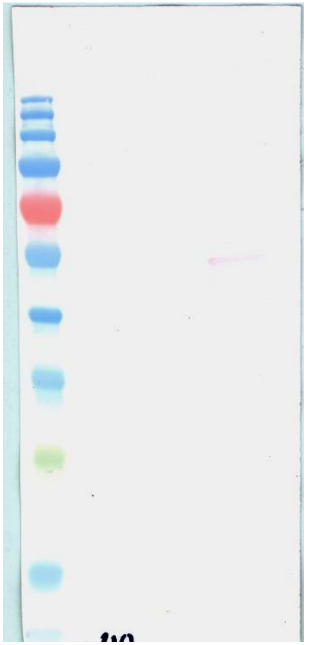

S1 S2 N

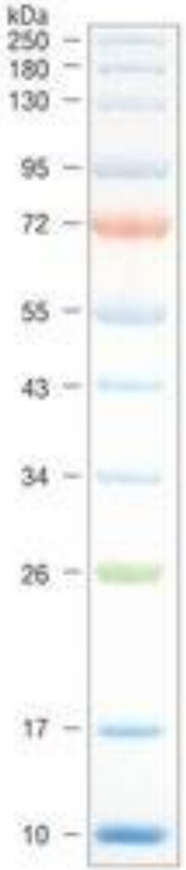

Supplement: Supplementary file 2 — Supplementary Information 2. [file 41598_2022_7263_MOESM2_ESM.pdf]
