# Supplementary material for: SARS-CoV-2 enzyme-linked immunosorbent assays as proxies for plaque reduction neutralisation tests
Source: Sci Rep. 2022 Mar 1;12:3351. doi: 10.1038/s41598-022-07263-8 (PMC8888744; doi:10.1038/s41598-022-07263-8)
Supplement: Supplementary file 3 — Supplementary Information 3. [file 41598_2022_7263_MOESM3_ESM.pdf]

Supplementary Data

SARS-CoV-2 enzyme-linked immunosorbent assays as proxies for plaque reduction neutralisation tests

Grant A Kay[1,2], MBChB, Sophie I Owen[1], MSc, Emanuele Giorgi[3], PhD, David J Clark[4], PhD, Christopher T Williams[1], MSc, Stefanie Menzies[1], PhD, Luis E Cuevas[1], PhD, Benedict M O Davies[4], MSc, Nicholas M Eckersley[4], MSc, Grant L Hughes[2], PhD, Daniela E Kirwan[4], MD, Sanjeev Krishna[4,5,6,7], ScD, Edward I Patterson[2,8], PhD, Tim Planche[4,5], MD, *Henry M Staines[4], DPhil, *Emily R Adams[1], PhD

[1]Centre for Drugs and Diagnostics, Liverpool School of Tropical Medicine, Liverpool, UK
[2]Departments of Vector Biology and Tropical Disease Biology, Centre for Neglected Tropical Diseases, Liverpool School of Tropical Medicine, Liverpool, UK
[3]Centre for Health Informatics Computing and Statistics, Lancaster University Medical School, Lancaster University, Lancaster, UK
[4]Centre for Diagnostics & Antimicrobial Resistance, Clinical Academic Group in Institute for Infection & Immunity, St George's University of London, London, UK
[5] St George's University Hospitals NHS Foundation Trust, UK
[6] Institut für Tropenmedizin, Universitätsklinikum Tübingen, Tübingen, Germany
[7] Centre de Recherches Médicales de Lambaréné, Gabon
[8] Department of Biological Sciences, Brock University, St. Catharines, Canada

Supplementary Data 1 – R-Script Ordinal Outcomes Model

```
rm(list=ls())
library(MASS)
cv <- read.csv("PRNT_ELISA paper 06.07.21.csv")
cv$SAMPLE.DAYS.POST.SYMP <- as.numeric(cv$SAMPLE.DAYS.POST.SYMP)
#cv$PRNT80[cv$PRNT80==1290] <- 1280
cv$ll <- cv$PRNT80
cv$ul <- NA
cv$ul[cv$ll>0 & cv$ll < 2560] <- cv$PRNT80[cv$ll>0 & cv$ll < 2560]*2
cv$ul[cv$ll==0] <- 20
cv$ul[cv$ll==2560] <- Inf

mle <- function(formula,return.se=TRUE,cv) {
  D <- model.matrix(formula,cv)
  p <- ncol(D)
  ll <- cv$ll
  ul <- cv$ul
  llik <- function(par) {
    beta <- par[1:p]
    mu <- exp(as.numeric(D%*%beta))
    sigma2 <- exp(par[p+1])
    mean.ln <- log(mu/sqrt(1+sigma2/(mu^2)))
    sd.ln <- sqrt(log(1+sigma2/(mu^2)))
    out <- sum(log(plnorm(ul,meanlog = mean.ln,sdlog = sd.ln)-
              plnorm(ll,meanlog = mean.ln,sdlog = sd.ln)))
  }
```

```
  start <- c(rep(0,p),1)
  estim <- nlminb(start,function(x)-llik(x))
  estim$reg.coef <- estim$par[1:p]
  estim$sigma2 <- exp(estim$par[p+1])
  estim$sigma <- sqrt(exp(estim$par[p+1]))
  names(estim$reg.coef) <- colnames(D)
  if(return.se) {
    library(numDeriv)
    H <- hessian(llik,estim$par)
    S <- solve(-H)
    se <- sqrt(diag(S))
    estim$covariance <- S
    estim$reg.coef.ci <- cbind(
      estim$reg.coef-qnorm(0.975)*se[1:p],
      estim$reg.coef+qnorm(0.975)*se[1:p])
    estim$sigma2.ci <- exp(cbind(estim$par[p+1]-qnorm(0.975)*se[p+1],
                             estim$par[p+1]+qnorm(0.975)*se[p+1]))
    estim$sigma.ci <- sqrt(estim$sigma2.ci)
  }
  return(estim)
}

str(cv)

# IgG__S1
fit_igg_s1_dps <-mle(~ 1+IgG_S1+SAMPLE.DAYS.POST.SYMP,cv=cv)
fit_igg_s1 <-mle(~ 1+IgG_S1,cv=cv)
1-pchisq(2*(fit_igg_s1$objective-fit_igg_s1_dps$objective),df=2)

# IgM__S1
fit_igm_s1_dps <-mle(~ 1+IgM_S1+SAMPLE.DAYS.POST.SYMP,cv=cv)
fit_igm_s1 <-mle(~ 1+IgM_S1,cv=cv)
1-pchisq(2*(fit_igm_s1$objective-fit_igm_s1_dps$objective),df=2)

# IgG__S2NP
fit_igg_s2_dps <-mle(~ 1+IgG_S2NP+SAMPLE.DAYS.POST.SYMP,cv=cv)
fit_igg_s2 <-mle(~ 1+IgG_S2NP,cv=cv)
1-pchisq(2*(fit_igg_s2$objective-fit_igg_s2_dps$objective),df=2)

# IgG__S2NP
fit_igm_s2_dps <-mle(~ 1+IgM_S2NP+SAMPLE.DAYS.POST.SYMP,cv=cv)
fit_igm_s2 <-mle(~ 1+IgM_S2NP,cv=cv)
1-pchisq(2*(fit_igm_s2$objective-fit_igm_s2_dps$objective),df=2)

which.min(c(fit_igg_s1$objective,
        fit_igm_s1$objective,
        fit_igg_s2$objective,
```

```
          fit_igm_s2$objective))

IgM.val <- quantile(cv$IgM_S1,c(0.25,0.5,0.975))
IgG.val <- quantile(cv$IgG_S1,c(0.25,0.5,0.975))

Sigma.par.sroot <- t(chol(fit_igm_s1$covariance))
n.sim <- 10000
par.sim.igm <- sapply(1:n.sim, function(i) fit_igm_s1$par+
                Sigma.par.sroot%*%rnorm(length(fit_igm_s1$par)))
par.sim.igg <- sapply(1:n.sim, function(i) fit_igg_s1$par+
                  Sigma.par.sroot%*%rnorm(length(fit_igm_s1$par)))

ll.set <- sort(unique(cv$ll))
ul.set <- sort(unique(cv$ul))
compute.probs <- function(beta,sigma2,x) {
  #IgG <- IgG.val[2]
  #beta <- fit2$reg.coef
  #sigma2 <- fit2$sigma2
  p <- length(beta)
  mu <- exp(sum(as.numeric(c(1,x)*beta)))
  mean.ln <- log(mu/sqrt(1+sigma2/(mu^2)))
  sd.ln <- sqrt(log(1+sigma2/(mu^2)))
  probs <- plnorm(ul.set,meanlog = mean.ln,sdlog = sd.ln)-
    plnorm(ll.set,meanlog = mean.ln,sdlog = sd.ln)
  return(probs)
}

n.titre <- length(unique(cv$PRNT80))
probs.samples.igm <- array(NA,dim=c(length(IgM.val),n.titre,n.sim))
for(i in 1:n.sim) {
  for(j in 1:length(IgM.val)) {
    p <- length(fit_igm_s1$par)-1
    beta.i <- par.sim.igm[1:p,i]
    sigma2.i <- exp(par.sim.igm[p+1,i])
    probs.samples.igm[j,,i] <- compute.probs(beta.i,sigma2.i,IgM.val[j])
  }
}

n.titre <- length(unique(cv$PRNT80))
probs.samples.igg <- array(NA,dim=c(length(IgG.val),n.titre,n.sim))
for(i in 1:n.sim) {
  for(j in 1:length(IgG.val)) {
    p <- length(fit_igg_s1$par)-1
    beta.i <- par.sim.igg[1:p,i]
    sigma2.i <- exp(par.sim.igg[p+1,i])
    probs.samples.igg[j,,i] <- compute.probs(beta.i,sigma2.i,IgG.val[j])
  }
```

```
}

prob.hat.igm <- apply(probs.samples.igm,c(1,2),mean)
prob.q025.igm <- apply(probs.samples.igm,c(1,2),function(x) quantile(x,0.025))
prob.q975.igm <- apply(probs.samples.igm,c(1,2),function(x) quantile(x,0.975))

prob.hat.igg <- apply(probs.samples.igg,c(1,2),mean)
prob.q025.igg <- apply(probs.samples.igg,c(1,2),function(x) quantile(x,0.025))
prob.q975.igg <- apply(probs.samples.igg,c(1,2),function(x) quantile(x,0.975))


pdf("First quartile IgM & IgG.pdf",width = 8,height = 8)
xlab <- c("<1:20",paste("1:",sort(unique(cv$PRNT80)[-1]),sep=""))
matplot(cbind(prob.hat.igg[1,],prob.q025.igg[1,],prob.q975.igg[1,],
          prob.hat.igm[1,],prob.q025.igm[1,],prob.q975.igm[1,]),
      type=c("b","l","l"),pch=20,col=c(1,1,1,2,2,2),
      lty=c("solid","dashed","dashed","solid","dashed","dashed"),
      axes=FALSE,ylab = "Probability",xlab="PRNT80",
      main="1st Quartile IgM & IgG")
axis(1,1:length(xlab),xlab)
axis(2,seq(0,1,0.05),seq(0,1,0.05))
legend(2,0.45,c("IgG S1","IgM S1"),col=c("black","red"),lty=1)
dev.off()

pdf("Median IgM & IgG.pdf",width = 8,height = 8)
xlab <- c("<1:20",paste("1:",sort(unique(cv$PRNT80)[-1]),sep=""))
matplot(cbind(prob.hat.igg[2,],prob.q025.igg[2,],prob.q975.igg[2,],
          prob.hat.igm[2,],prob.q025.igm[2,],prob.q975.igm[2,]),
      type=c("b","l","l"),pch=20,col=c(1,1,1,2,2,2),
      lty=c("solid","dashed","dashed"),
      axes=FALSE,ylab = "Probability",xlab="PRNT80",
      main="Median IgM & IgG")
axis(1,1:length(xlab),xlab)
axis(2,seq(0,1,0.05),seq(0,1,0.05))
legend(2,0.3,c("IgG S1","IgM S1"),col=c("black","red"),lty=1)
dev.off()

pdf("Third Quartile IgM & IgG.pdf",width = 8,height = 8)
xlab <- c("<1:20",paste("1:",sort(unique(cv$PRNT80)[-1]),sep=""))
matplot(cbind(prob.hat.igg[3,],prob.q025.igg[3,],prob.q975.igg[3,],
          prob.hat.igm[3,],prob.q025.igm[3,],prob.q975.igm[3,]),
      type=c("b","l","l"),pch=20,col=c(1,1,1,2,2,2),
      lty=c("solid","dashed","dashed"),
      axes=FALSE,ylab = "Probability",xlab="PRNT80",
      main="3rd Quartile IgM & IgG")
axis(1,1:length(xlab),xlab)
axis(2,seq(0,1,0.05),seq(0,1,0.05))
```

```
legend(2,0.5,c("IgG S1","IgM S1"),col=c("black","red"),lty=1)
dev.off()

IgM.set <- seq(-0.352,2.337,length=1000)
IgG.set <- seq(-0.352,2.337,length=1000)

mean.samples.igm <- matrix(NA,nrow=length(IgM.set),ncol=n.sim)
for(i in 1:n.sim) {
  p <- length(fit_igm_s1$par)-1
  beta.i <- par.sim.igm[1:p,i]
  mean.samples.igm[,i] <- exp(beta.i[1]+beta.i[2]*IgM.set)
}

mean.samples.igg <- matrix(NA,nrow=length(IgG.set),ncol=n.sim)
for(i in 1:n.sim) {
  p <- length(fit_igg_s1$par)-1
  beta.i <- par.sim.igg[1:p,i]
  mean.samples.igg[,i] <- exp(beta.i[1]+beta.i[2]*IgM.set)
}

mean.hat.igm <- apply(mean.samples.igm,1,mean)
mean.q025.igm <- apply(mean.samples.igm,1,function(x) quantile(x,0.025))
mean.q975.igm <- apply(mean.samples.igm,1,function(x) quantile(x,0.975))

mean.hat.igg <- apply(mean.samples.igg,1,mean)
mean.q025.igg <- apply(mean.samples.igg,1,function(x) quantile(x,0.025))
mean.q975.igg <- apply(mean.samples.igg,1,function(x) quantile(x,0.975))

pdf("Predicted_nAB_vs_IgM_IgG.pdf",width = 8,height = 8)
xlab <- c("<1:20",paste("1:",sort(unique(cv$PRNT80)[-1]),sep=""))
matplot(IgM.set,cbind(mean.hat.igg,mean.q025.igg,mean.q975.igg,
                    mean.hat.igm,mean.q025.igm,mean.q975.igm),
       type=c("l","l","l"),pch=20,col=c(1,1,1,2,2,2),
       lty=c("solid","dashed","dashed"),
       ylab = "nAB",xlab="IgM")
legend(0,1500,c("IgG S1","IgM S1"),col=c("black","red"),lty=1)
dev.off()


round(rbind(cbind(fit_igm_s1$reg.coef,fit_igm_s1$reg.coef.ci),
      sqrt(c(fit_igm_s1$sigma2,fit_igm_s1$sigma2.ci))),3)
```
